# Increased CD14^+^HLA-DR^−/low^ Myeloid-Derived Suppressor Cells Correlate With Disease Severity in Systemic Lupus Erythematosus Patients in an iNOS-Dependent Manner

**DOI:** 10.3389/fimmu.2019.01202

**Published:** 2019-05-29

**Authors:** Zhitao Wang, Fengfeng Zhu, Jiyu Wang, Qianshan Tao, Xuanxuan Xu, Huiping Wang, Shudao Xiong, Yiping Wang, Zhimin Zhai

**Affiliations:** ^1^Department of Hematology, The Second Affiliated Hospital of Anhui Medical University, Hefei, China; ^2^Department of Rheumatology, The Second Affiliated Hospital of Anhui Medical University, Hefei, China; ^3^Centre for Transplantation and Renal Research, Westmead Millennium Institute, The University of Sydney, Sydney, NSW, Australia

**Keywords:** myeloid-derived suppressor cells, SLE, iNOS, disease severity, immunosuppression

## Abstract

Myeloid-derived suppressor cells (MDSCs) comprise of a population of cells, which suppress the innate and adaptive immune system via different mechanisms. MDSCs are accumulated under pathological conditions. The present study aimed to clarify the pathological role of MDSCs in systemic lupus erythematosus (SLE) patients. Consequently, the level of circulating M-MDSCs was significantly increased in newly diagnosed SLE patients as compared to healthy controls. An elevated level of M-MDSCs was positively correlated with the disease severity in SLE patients and an immunosuppressive role was exerted in an iNOS-dependent manner. The decrease in the number of M-MDSCs after therapy rendered them as an indicator for the efficacy of treatment. These results demonstrated that M-MDSCs participated in the pathological progress in SLE patients. Thus, MDSCs are attractive biomarkers and therapeutic targets for SLE patients.

## Introduction

Systemic lupus erythematosus (SLE) is one of the most common autoimmune diseases (1). The main manifestations include the imbalance of peripheral immune tolerance to autoantigens. The excessive activation of T cells and the production of a large number of autoantibodies due to the hyperfunction of B cells disrupt the steady state of pro-inflammatory and anti-inflammatory factors as well as the accumulation of large amounts of immune complexes and inflammatory damage to multiple organs and tissue systems (2). The equilibrium in the immune system and its strict control by regulatory mechanisms is a critical issue in SLE. Interestingly, several individual cell subsets or molecules are involved in the pathogenesis of SLE. However, the specific pathogenesis is not yet clearly understood.

Myeloid-derived suppressor cells (MDSCs) are a group of immature cells that can inhibit the immune response, thereby promoting the occurrence and progress of tumors as described previously (3). MDSCs can promote tumor metastasis and angiogenesis by inhibiting the immune response and T cell proliferation (4). Also, MDSCs express Gr-1 and CD11b surface molecular markers that are divided into two types: granulocytic and monocytic phenotypes (5, 6). Anti-Gr-1 monoclonal antibody contains two molecules, Ly6G and Ly6C. In mice, MDSCs are specified as CD11b^+^Gr-1^+^Ly6G^+^ granulocytic MDSCs (G-MDSCs) and CD11b^+^Gr-1^+^Ly6C^+^ monocytic MDSCs (M-MDSCs) according to the phenotype (7, 8). Nonetheless, an unequivocal method for phenotyping the human MDSCs for the human equivalent of Gr-1 is lacking. G-MDSCs are primarily described as CD11b^+^CD33^+^CD15^+^HLA-DR^+^, whereas M-MDSCs are mainly described as CD11b^+^CD33^+^CD14^+^HLA-DR^low/−^ or CD14^+^HLA-DR^low/−^ in humans (9–11). MDSCs inhibit the immune response through different pathways, including direct and indirect contact. For example, MDSCs secrete a variety of immunomodulatory factors to exert an inhibitory effect, including inducible nitric oxide synthase (iNOS) and arginase-1 (Arg-1) (12, 13). Previous studies have shown that MDSCs can be accumulated by several cytokines *in vitro*, for example granulocyte macrophage colony stimulating factor (GM-CSF) and interleukin-6 (IL-6) (14).

In recent years, the role of MDSCs has attracted increasing attention with respect to autoimmune diseases (15). These cells are involved in the occurrence and development of a variety of autoimmune diseases, including rheumatoid arthritis (RA) (16), type I diabetes (TID) (17), multiple sclerosis (MS) (18), and inflammatory bowel disease (IBD) (19). Moreover, previous studies mainly focused on mouse models, and only a few studies were carried out in patients (20–23). Previously, Wu et al. found active SLE patients had a significant increase in HLA-DR^−^CD11b^+^CD33^+^ MDSCs, including both CD14^+^CD66b^−^ monocytic and CD14^−^CD66b^+^ granulocytic MDSCs in the peripheral blood as compared to healthy controls (24). The frequency of M-MDSCs was positively correlated with disease severity and MDSCs were pathogenic for SLE by induction of Th17 cells. However, the role of MDSCs in the pathogenesis of SLE needs further investigation.

In this study, we evaluated the correlation of another immunophenotype M-MDSCs (CD14^+^HLA-DR^−/low^MDSCs) with clinical parameters and the possible mechanisms in pathogenesis of SLE patients. We found that the level of circulating M-MDSCs was significantly increased and positively correlated with the disease activity in newly diagnosed SLE patients. Additionally, the elevated level of M-MDSCs exerted an immunosuppressive role in an iNOS-dependent manner. The current study provides a new theoretical basis for the pathogenic role of MDSCs in SLE and puts forth new therapeutic targets for the clinical treatment of SLE patients.

## Materials and Methods

### Patients and Samples

A total of 32 patients, diagnosed with SLE from May 2016 to March 2018 at the Second Affiliated Hospital, Anhui Medical University, China, were enrolled in this study. Patients with comorbidities that might affect their immune status with respect to inflammation or tumors were excluded from the study. The detailed clinical data of the patients are shown in Table 1. All patients with SLE fulfilled the revised disease criteria of the American College of Rheumatology (25). The disease activity was assessed based on the SLE Disease Activity Index (SLEDAI) (26). According to the SLEDAI score, SLE patients were divided into two groups: active group (*n* = 18, SLEDAI > 9) and inactive group (*n* = 14, SLEDAI ≤ 9). The 32 newly diagnosed SLE patients were administered glucocorticoid, cyclophosphamide, or methotrexate therapy according to the patient's condition. The samples from these patients were collected prior to any treatment and after at least 3 months of treatment. Moreover, the samples were evaluated within 6 h of collection.

**Table 1:** Characteristics of healthy donors and SLE patients.

**State of disease at sample draw**	**No. of patients**	**Average age (range)**
Newly diagnosed	32	27.7 (18–36)
Gender		
Male	6	28.6 (26–33)
Female	26	27.5 (18–36)
Lupus nephritis		
YES	12	27.8 (19–36)
NO	20	27.7 (18–33)
Active disease		
YES	18	27.2 (18–36)
NO	14	28 (21–34)
Anti-dsDNA antibody		
Positive	20	29.5 (20–36)
Negative	12	24.8 (18–32)

The study protocol was approved by the Ethics Committee of Anhui Medical University. Written informed consents were obtained from all patients and volunteers.

### Flow Cytometric Analysis

The following monoclonal antibodies were purchased from Beckman Coulter Immunology (Miami, FL, USA): APC-labeled CD14 (clone RMO52), ECD-labeled HLA-DR (clone Immu-375), FITC-labeled CD14 (clone 116), PE-labeled HLA-DR (clone B8.12.2), and PE-labeled anti-CD4 (clone 13B8.2). Peripheral blood mononuclear cells (PBMCs) were stratified on Ficoll-Hypaque (Amersham Biosciences, Sweden) and separated by centrifugation for 25 min (500 × g). Subsequently, PBMCs were collected and washed with phosphate-buffered saline (PBS). After washing, 100 μL PBMCs was incubated with CD antibody and analyzed by flow cytometry (FC500 MPL, Beckman Coulter), and EXPO 32 Multicomp software was used for data acquisition and analysis. All the samples were matched with the same type of antibody as a control.

### Identification of MDSCs

PBMCs were separated from 5 newly diagnosed SLE patients (SLEDAI > 10) and stained with antibodies to human CD4, CD14, and HLA-DR. In suppressive assays, autologous CD4^+^T cells, CD14^+^HLA-DR^−/low^MDSCs, and CD14^+^HLA-DR^+^ cells were sorted using MoFlo XDP cell sorter (Beckman Coulter). The purity of the cells was >95% after sorting. CD4^+^T cells were labeled with carboxy fluorescein succinimidyl ester (CFSE) (0.5 μmol/L) according to the manufacturer's instructions (Invitrogen, Carlsbad, CA, USA). CFSE-labeled CD4^+^T cells were washed and co-cultured with M-MDSCs and CD14^+^HLA-DR^+^cells in a ratio of 1:1 in 96-well plates (Wuxi Nest Biotechnology Co., Ltd, Wuxi, China) in complete RPMI 1640 medium with 10% fetal bovine serum (Gibco, Carlsbad, CA, USA). CD4^+^T cells were cultured separately and used as positive control. All cells were incubated with anti-CD3 (2 μg/mL), anti-CD28 (5 μg/mL). Following 3 days of co-culture, the rate of proliferation of CD4^+^T cells was determined by FACS analysis. The concentration of IFN-γ was measured by ELISA Kit (R&D systems, Minneapolis, MN, USA) according to the manufacturer's instructions.

### RNA Isolation and RT-PCR

Total RNA was extracted from PBMCs using TRIzol reagent (Invitrogen). cDNA was synthesized by reverse transcription using oligo(dT). RT-PCR for *iNOS* and a reference gene (β*-actin*) was performed in the real-time PCR model 7300 (Applied Biosystems, USA) using Power SYBR Green PCR Master Mix (Applied Biosystems) according to the manufacturer's instructions. The expression of the target gene was normalized against that of β*-actin*. The primer sequences used were as follows: (5′-CTTTCCAAGACACACTTCACCA-3′) and reverse (5′-TATCTCCTTTGTTAC CGCTTCC-3′) for *iNOS*; forward (5′-TGGCACCCAGCACAATGAA-3′) and reverse (5′-CTAAGTCATAGTCCGCT AGAAGCA-3′) for β*-actin*.

### ELISA Assay

Plasma was collected at the same time when PBMCs were isolated from SLE patients and healthy controls. Plasma concentrations of IL-6, GM-CSF, and Arg-1 were measured by ELISA Kit (R&D systems).

### Cell Culture and Cytokine Induction

PBMCs (1 × 10^6^) from healthy controls were incubated with plasma (500 μL) from 5 newly diagnosed SLE patients (SLEDAI > 10) and 5 healthy controls in 24-well-plates in triplicate (Wuxi Nest Biotechnology) for 72 h. Recombinant human granulocyte-macrophage colony stimulating factor (rhGM-CSF, 10 ng/mL; Sigma) was added to the mixture to enhance cell viability (14, 27). The cells were cultured at 37°C in a humidified CO_2_ incubator.

### Cell Isolation and Sorting

After 72 h, all cells were collected from PBMC cultures. Autologous CD4^+^T Cells, CD14^+^HLA-DR^−/low^MDSCs, and CD14^+^HLA-DR^+^ cells were sorted as described above.

### Assay for T Cell Proliferation

CD4^+^T cells were labeled with 0.5 μmol/L CFSE. Subsequently, the cells were washed and co-cultured at different ratios with M-MDSCs (16:1, 4:1, 1:1) and CD14^+^HLA-DR^+^cells (1:1) in 96-well plates in complete RPMI 1640 medium with 10% fetal bovine serum. CD4^+^T cells cultured alone were used as positive control. All cells were incubated with anti-CD3 (2 μg/mL), anti-CD28 (5 μg/mL). After 3 days of co-culture, the proliferation of CD4^+^T cells was determined by FACS analysis. Additionally, the inhibitors of candidate suppressive molecules were added at the following final concentrations in the co-culture of CD4^+^T cells and M-MDSCs (1:1): 500 μmol/L Nx-Hydroxy-nor-L-arginine, diacetate salt (nor-NOHA, Calbiochem, Darmstadt, Germany), and 500 μmol/L NG-Methyl-L-arginine acetate salt (L-NMMA, Sigma-Aldrich). Supernatants were stored at −80°C until further use. The concentration of IFN-γ was measured by ELISA Kit according to the manufacturer's instructions.

### Statistical Analysis

SPSS17.0 software was used for statistical analysis (SPSS Inc., Chicago, IL, USA). Paired and unpaired samples were tested for statistical significance by non-parametric Mann–Whitney *U* test and parametric Student's *t*-test. Univariate analysis of variance (ANOVA) and LSD *t*-test were used to compare the levels of M-MDSCs in different groups. Pearson's coefficient test was employed for correlation analysis. In all the analyses, *P* < 0.05 was considered significant.

## Results

### Expression of M-MDSCs in SLE Patients Is Increased

**Figure 1:**
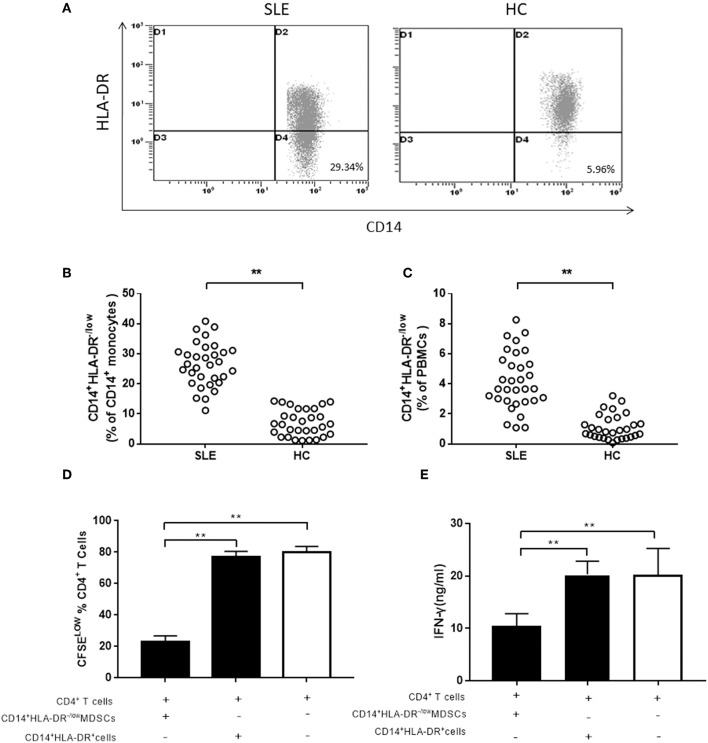
The expression of M-MDSCs in SLE patients. **(A)** Representative flow cytometric speckle maps showed the expression of M-MDSCs in newly diagnosed SLE patients and healthy controls. **(B)** M-MDSCs levels in newly diagnosed SLE patients and healthy controls (in CD14^+^ monocytes). **(C)** M-MDSCs levels in newly diagnosed SLE patients and healthy controls (in PBMCs). **(D)** MDSCs had a significantly immunosuppressive activity on CD4^+^T cells as compared to the CD14^+^HLA-DR^+^ cells from active SLE patients. The proliferation of CD4^+^T cells was measured by dilution of CFSE staining intensity using flow cytometry. **(E)** IFN-γ-producing was also significantly decreased in CD4^+^T cells co-cultured with M-MDSCs. Each point represents an individual. The horizontal bar represents the average. **P < 0.01.

Hitherto, there is no uniform standard for cell surface markers of human MDSCs. Although G-MDSCs are prevalent, the M-MDSCs exert a stronger immunosuppressive effect than G-MDSCs (28). Peripheral CD14^+^HLA-DR^−/low^ cells were characterized as M-MDSCs in this study. We investigated the frequency of M-MDSCs in 32 newly diagnosed SLE patients, as well as 30 age- and sex-matched healthy controls. In CD14^+^ monocytes and PBMCs, the frequency of M-MDSCs was significantly higher in SLE patients than that in healthy controls (Figures 1A–C). Next, the immunosuppressive ability of CD14^+^HLA-DR^−/low^ cells was investigated, which was the central element to identify MDSCs. Compared to the CD14^+^HLA-DR^+^ cells, M-MDSCs significantly inhibited the proliferation of CD4^+^T cells (Figure 1D). Moreover, the ability of CD4^+^T cells co-cultured with M-MDSCs to produce IFN-γ was significantly decreased as compared to CD14^+^HLA-DR^+^ cells (Figure 1E).

### Expansion of M-MDSCs Is Positively Correlated With Disease Severity and Response to Treatment in SLE Patients

**Figure 2:**
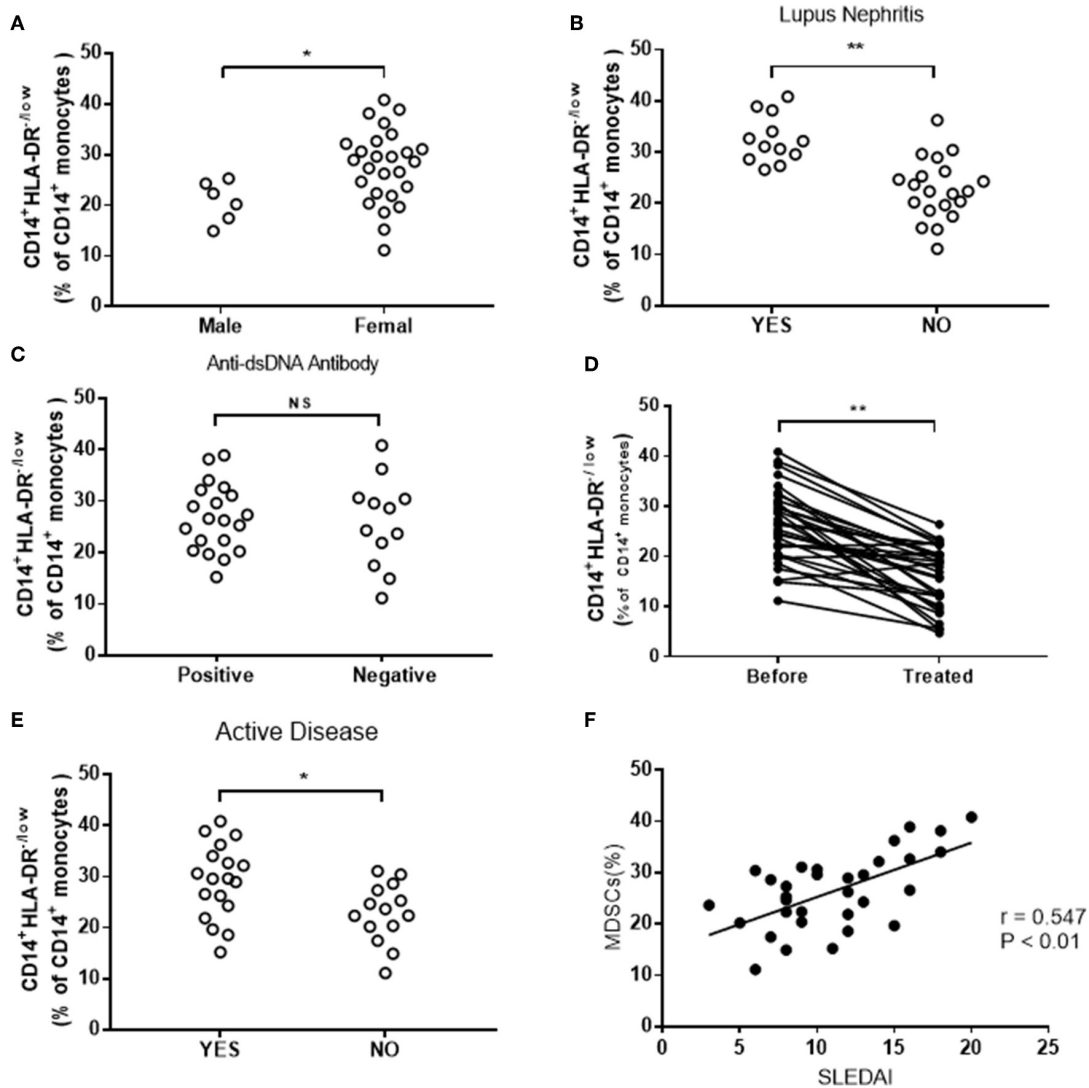
Clinical correlation of M-MDSCs in SLE patients. **(A)** The frequency of M-MDSCs in female patients was significantly higher than that in male patients. **(B)** The frequency of M-MDSCs increased in patients with lupus nephritis. **(C)** No significant difference was detected in M-MDSCs between anti-dsDNA-positive and anti-dsDNA-negative groups (YES, lupus nephritis; NO, non-lupus nephritis). **(D)** The frequency of M-MDSCs was significantly decreased after treatment. **(E)** The level of M-MDSCs in patients with active SLE was higher than that in patients with inactive SLE (YES, active SLE patients; NO, inactive SLE patients). **(F)** M-MDSCs was positively correlated with the score of SLEDAI in SLE patients. Each point represents an individual. The horizontal bar represents the average. *P < 0.05; **P < 0.01.

Further studies revealed that the frequency of M-MDSCs was corrected with the severity of the disease. In present study, we grouped all SLE patients by types of clinicopathological factors, which indicated disease progression, including gender, lupus nephritis, anti-dsDNA antibody, and disease activity. Higher levels of M-MDSCs were found in female patients as compared to male patients (Figure 2A). The frequency of M-MDSCs in patients with lupus nephritis is higher than that in patients with normal renal function (Figure 2B). However, no significant difference was found between anti-dsDNA positive and anti-dsDNA-negative groups (Figure 2C). The frequency of M-MDSCs was significantly decreased as compared to the same patients before treatment (Figure 2D).

In addition, the correlation between M-MDSCs and SLEDAI was analyzed. We found that M-MDSCs levels were higher in the active group than in the inactive group (Figure 2E). Furthermore, the score of SLEDAI was positively correlated with the frequency of M-MDSCs (*r* = 0.547, *P* < 0.01, Figure 2F).

### M-MDSCs-Associated Immunosuppressive Factors in SLE Patients

**Figure 3:**
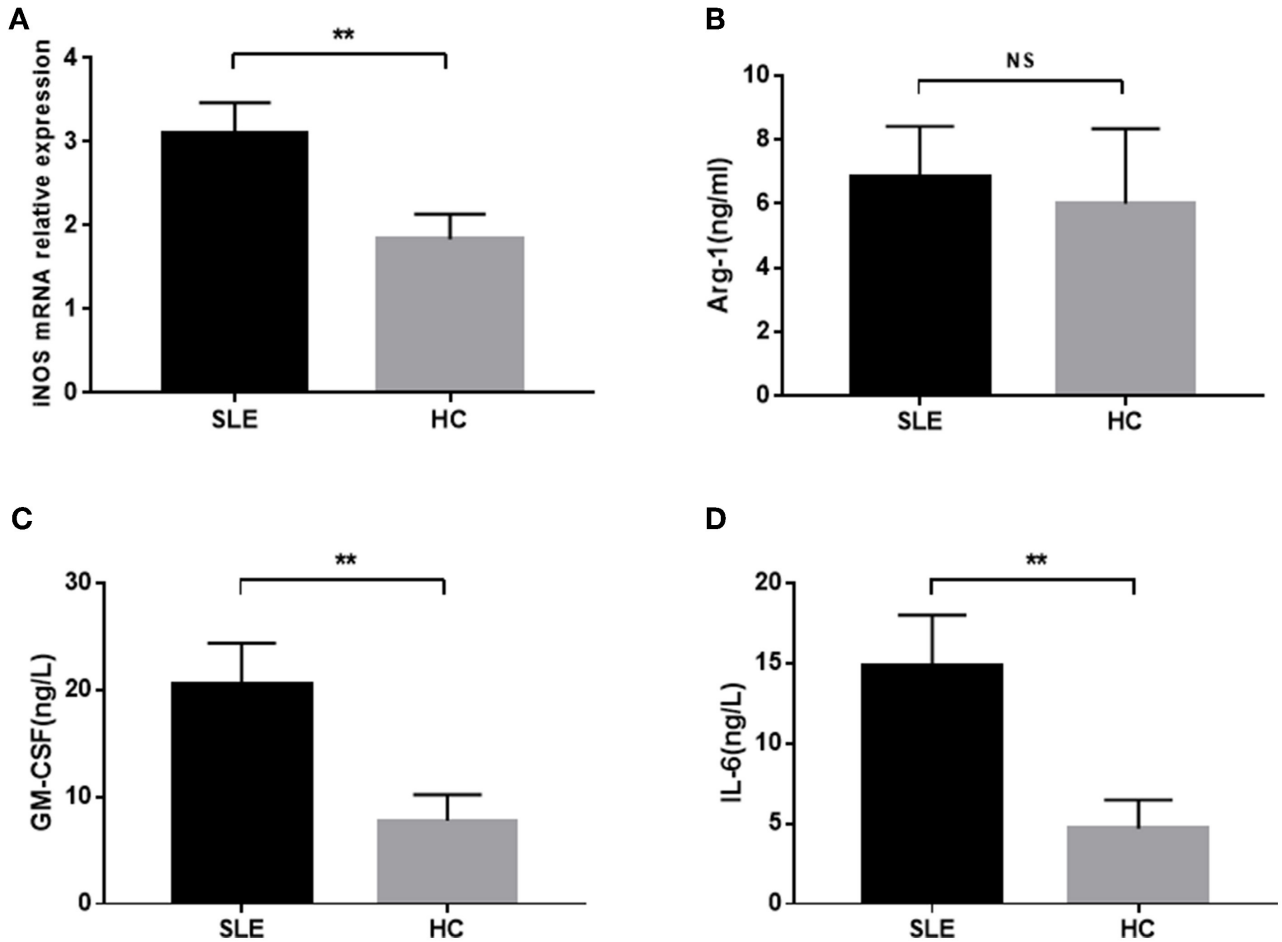
The concentration of *iNOS* mRNA, Arg-1, GM-CSF, and IL-6 in newly diagnosed SLE patients. **(A)** The level of *iNOS* mRNA in SLE patients was significantly higher than that in healthy controls. **(B)** Plasma level of Arg-1 was not significantly elevated in SLE patients. **(C,D)** The plasma levels of GM-CSF and IL-6 in SLE patients were significantly higher than those in healthy controls. **P < 0.01.

M-MDSCs secrete a variety of cytokines and enzymes, including iNOS and Arg-1, to exert the immunosuppressive effect. Next, we detected the levels of MDSCs-associated enzymes in SLE patients. A significant increased level of *iNOS* mRNA was observed between SLE patients and healthy donors (Figure 3A). Also, a higher concentration of Arg-1 was found in SLE patients as compared to healthy donors, albeit not significantly (Figure 3B).

Previous studies have shown that M-MDSCs could be induced by specific cytokines, GM-SCF and IL-6, in the inflammatory state (14). Thus, we detected the levels of GM-SCF and IL-6 in the peripheral blood of SLE patients and found that they were significantly increased in the peripheral blood of newly diagnosed SLE patients (Figures 3C,D).

### Plasma From SLE Patients Induce the Expansion of M-MDSCs *in vitro*

**Figure 4:**
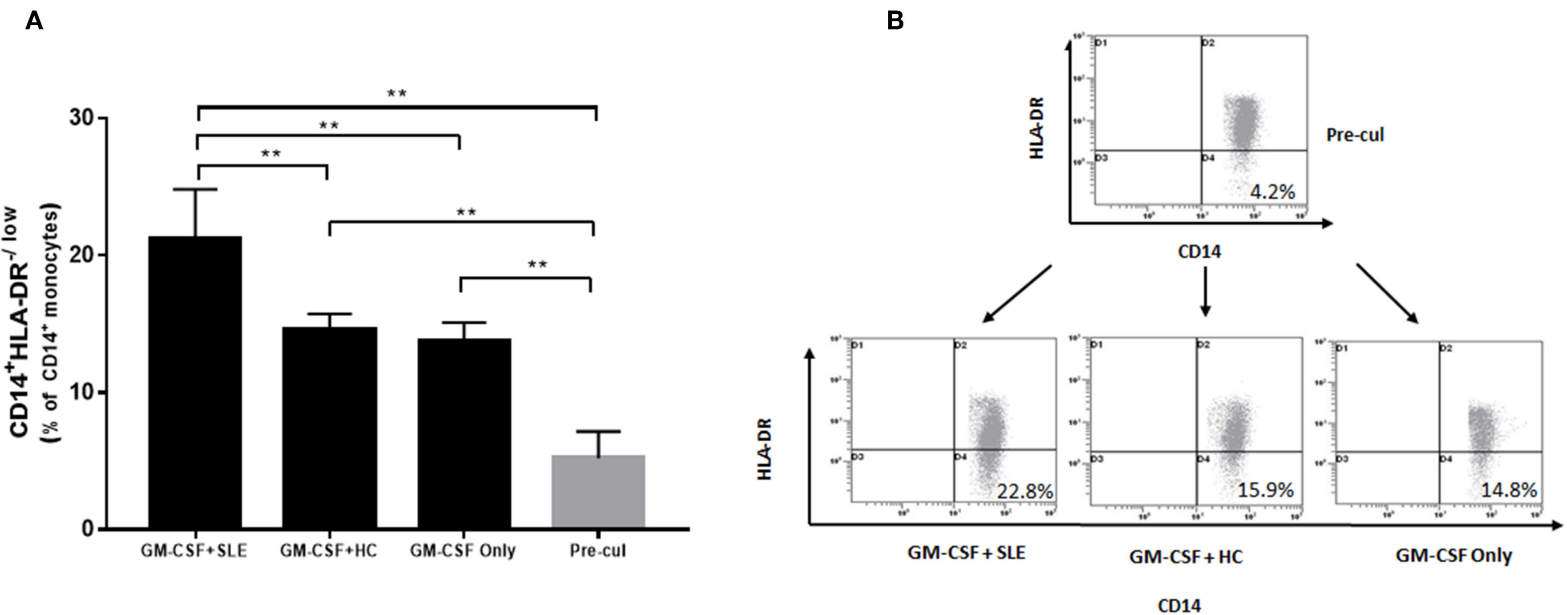
Human PBMCs isolated from healthy controls were incubated with plasma from 5 newly diagnosed SLE patients and healthy controls. **(A)** Plasma from newly diagnosed SLE patients could induce a significant proliferation of M-MDSCs *in vitro* as compared to the plasma from healthy controls as well as with pre-culture (*P* < 0.01). **(B)** Representative flow cytometric dot plots showed the proliferation of M-MDSCs. *Pre-cul, pre-culture*; **P < 0.01.

Furthermore, we determined the putative mechanisms of proliferation of M-MDSCs *in vitro*. Human PBMCs were isolated from 5 healthy volunteer controls and isolated by differential density gradient separation. An equivalent of 1 × 10^6^ PBMCs from healthy controls was incubated with plasma (500 μL) from newly diagnosed SLE patients (SLEDAI > 10) and healthy controls in 24-well plates for 72 h *in vitro*. As shown in Figures 4A,B, the percentage of M-MDSCs co-cultured with plasma from SLE patients was significantly increased as compared to the plasma from healthy controls as well as with pre-culture (*P* < 0.01).

### Immunosuppressive Effect of Induced M-MDSCs on Autologous CD4^+^T Cells

**Figure 5:**
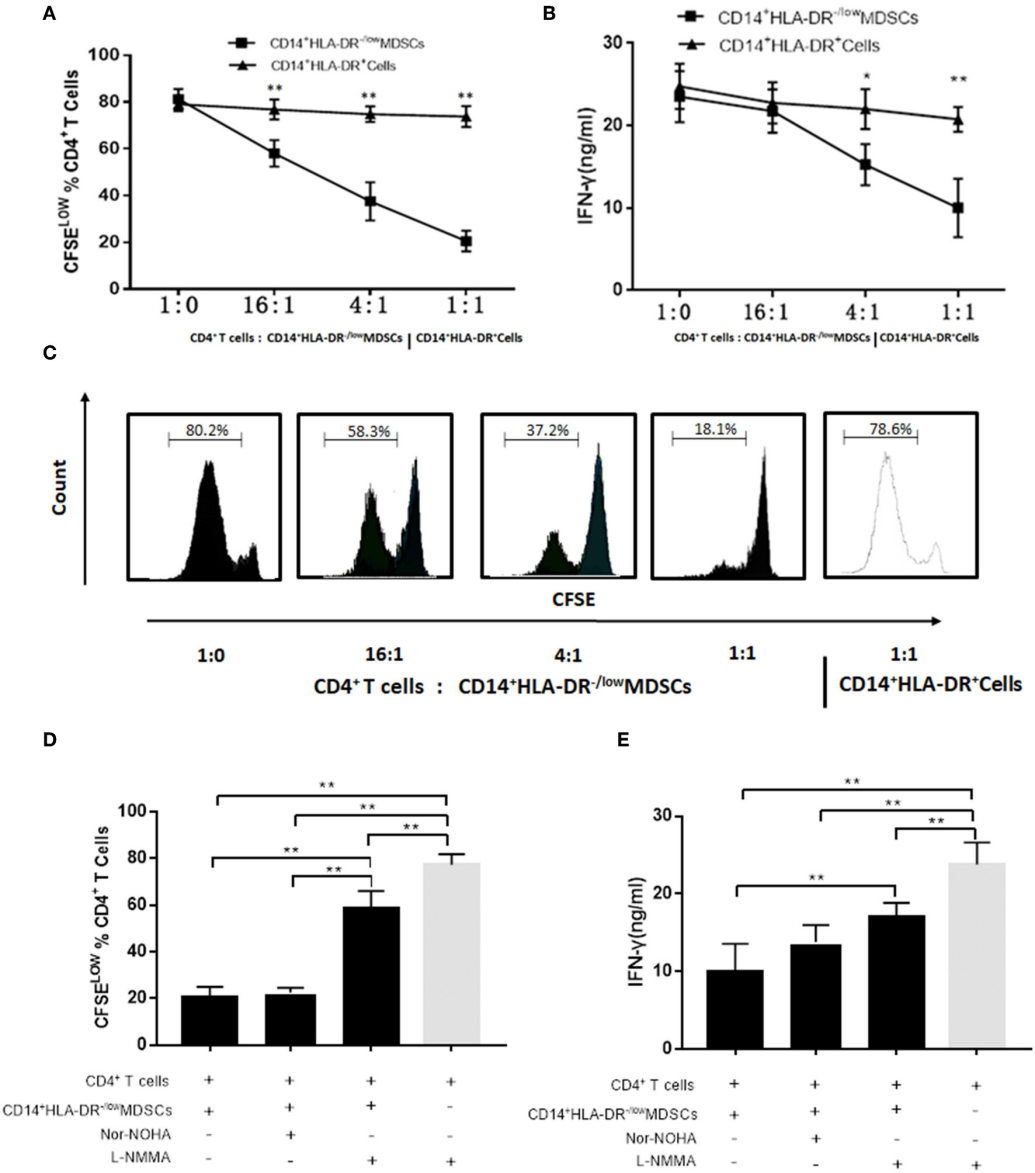
Effects of M-MDSCs on the proliferation of autologous T cells and production of IFN-γ. **(A)** Plasma-induced MDSCs had a significant immunosuppressive activity on CD4^+^ T cells in a dose-dependent manner as compared to the CD14^+^HLA-DR^+^ cells. The proliferation of CD4^+^T cells was measured by dilution of CFSE staining intensity using flow cytometry. **(B)** IFN-γ-production was also significantly decreased in CD4^+^T cells co-cultured with M-MDSCs. **(C)** Representative flow cytometry flow histograms demonstrated the immunosuppressive activity of M-MDSCs and CD14^+^HLA-DR^+^cells. **(D,E)** Effects of iNOS and Arg-1 inhibitors on the M-MDSC suppression with respect to CD4^+^T cell proliferation and IFN-γ production. *Ctrl, control*; *P < 0.05; **P < 0.01.

Firstly, the immunosuppressive activity of M-MDSCs on autologous T cell proliferation and IFN-γ production was evaluated. Next, the suppression of T cell proliferation was assessed by CFSE dilution after a 3-days co-culture of MDSCs and compared to that elicited by HLA-DR^+^ control cells. M-MDSCs significantly inhibited the proliferation of CD4^+^T cells in a dose-dependent manner as compared to the CD14^+^HLA-DR^+^ cells (Figures 5A,C). Moreover, the ability of CD4^+^T cells co-cultured with M-MDSCs to produce IFN-γ was significantly decreased as compared to the CD14^+^HLA-DR^+^ cells (Figure 5B).

We found that the level of iNOS mRNA was significantly increased in SLE patients as compared to the healthy donors as described above. We hypothesized that M-MDSCs play an immunosuppressive role through iNOS in SLE patients. As we estimated, the proliferation of CD4^+^T cells (Figure 5D) and production of IFN-γ (Figure 5E) were significantly restored by L-NMMA, which is the specific inhibitor of iNOS. Moreover, this phenomenon was not found in the addition of Arg-1 inhibitor Nor-NOHA, which further confirmed that the production of iNOS was the main method to exert an immunosuppressive effect on M-MDSCs in SLE patients (Figures 5D,E).

## Discussion

Myeloid suppressor cells are immature populations of immunosuppressive cells derived from bone marrow. Initially, the number of MDSCs expanded normally in the tumor, which inhibited the T cell immune response and promoted tumor progression (29). In recent years, the research field of MDSCs gradually involved with respect to autoimmune diseases. Accumulating evidence indicated that MDSCs participated in the inflammatory immune response to autoimmune diseases (30). Different from the role of MDSCs in promoting tumor progression, the role of MDSCs in autoimmune diseases is not clear. In several autoimmune disease mouse models, MDSCs increased significantly (31). However, MDSCs could not effectively alleviate the disease and also promote the progression of the disease. This contradicts the theory that MDSCs has immunosuppressive function and should inhibit the progression of autoimmune diseases, and hence, it role in autoimmune diseases needs to be further clarified.

In this study, we evaluated the impact of M-MDSCs on clinical parameters in SLE patients. As described above, validated unique phenotypic markers for MDSCs are yet lacking. The immunosuppressive effect is a critical feature in the identification of MDSCs (32). Firstly, the immunosuppressive activity was investigated in CD14^+^HLA-DR^−/low^ cells from the peripheral blood of SLE patients, which was the central element to identify MDSCs. We evaluated the immunosuppressive activities of M-MDSCs on autologous T cell proliferation and IFN-γ production. We found that M-MDSCs significantly suppressed the proliferation of CD4^+^T cells as compared to the CD14^+^HLA-DR^+^ cells. Moreover, the production of IFN-γ was significantly decreased in CD4^+^T cells co-cultured with M-MDSCs. Strikingly, M-MDSCs exert a strong suppressive ability on T cell proliferation and IFN-γ production; however, this ability was not found in CD14^+^HLA-DR^+^ cells.

A significantly higher level of M-MDSCs was detected as compared to healthy controls. This phenomenon was in agreement with previous reports that MDSCs were involved in the pathological progress in SLE patients (23, 24). The frequency of M-MDSCs was increased markedly in female and lupus nephritis patients. Also, a positive correlation was established between the frequency of MDSCs and the disease activity in newly diagnosed SLE patients. Furthermore, the frequency of M-MDSCs decreased significantly after treatment. This observation indicated that the level of M-MDSCs was correlated with disease severity. The decrease in the frequency of M-MDSCs after treatment was due to the reduction in disease activity. Consequently, significant changes in the frequency of M-MDSCs after treatment can be used to reflect the efficacy of treatment in SLE patients. This phenomenon indicated that MDSC expansion could be recognized as a major pathophysiological feature in human SLE patients.

MDSCs inhibit immune system through several different mechanisms (33). It is also speculated that MDSCs inhibit the immune cells by direct contact between cells or indirectly by inducing the production of Tregs and the secretion of a variety of cytokines (34); for example, iNOS, Arg-1, ROS, and IL-10. G-MDSCs inhibit the T cell function by regulating ROS, while M-MDSCs inhibit the T cell function through iNOS and Arg-1 (35, 36). In the present study, the level of iNOS mRNA was significantly increased in newly diagnosed SLE patients. This indicated that MDSCs were an abundant source of iNOS production. MDSCs exerted immunosuppression in an iNOS-dependent manner.

Tumor cells and tumor-derived factors (such as GM-CSF and IL-6) can induce the accumulation of MDSCs in PBMCs from healthy donors (37, 38). GM-CSF and IL-6 are vital pro-inflammatory cytokines in human immune regulation (39, 40). Interestingly, the plasma concentration of GM-CSF and IL-6 was significantly increased in newly diagnosed SLE patients. Next, we determined the possible mechanisms underlying the *in vitro* proliferation of M-MDSCs. Human PBMCs isolated from healthy volunteers were incubated with plasma from newly diagnosed SLE patients and healthy volunteer controls. The plasma from newly diagnosed SLE patients could induce the proliferation of MDSCs *in vitro*. This phenomenon suggested that immune tolerance abnormalities occurred in SLE patients. A large number of activated T and B cells secreted inflammatory cytokines, such as GM-CSF and IL-6. MDSCs could be induced by these inflammatory cytokines in SLE patients.

Furthermore, MDSCs are defined by surface markers in conjunction with suppressive function (32). The immunosuppressive activities and mechanisms of plasma-induced M-MDSCs were evaluated on autologous T cell proliferation and IFN-γ production. M-MDSCs were found to significantly suppress the proliferation of CD4^+^T cells in a dose-dependent manner as compared to the CD14^+^HLA-DR^+^ cells. Moreover, IFN-γ-production was significantly decreased in CD4^+^T cells co-cultured with M-MDSCs. Also, the proliferation of T cells and production of IFN-γ were significantly restored by L-NMMA, which was the specific inhibitor of iNOS. M-MDSCs induced by plasma from SLE patients mediated a potent suppression of autologous T cell proliferation and IFN-γ production. We hypothesized that the high expression of MDSCs exerts immunosuppressive effect and mediates the abnormal immune tolerance of SLE through iNOS pathway. However, suppressing the abnormal hyperactivity of immune function of SLE is not sufficient. The adoptive infusion of exogenous MDSCs might alleviate the condition of SLE patients (41).

In summary, elevated MDSCs were positively correlated with disease activity in SLE patients. MDSCs were pathogenic for SLE patients. The current data highlights that MDSCs are immunosuppressive in SLE patients in an iNOS-dependent manner. The number of M-MDSCs decreased after treatment, suggesting that these cells can be used as one of the indicators to judge the treatment effect. Briefly, MDSCs are critical immunosuppressor agents during SLE progression and might represent effective therapeutic targets for the treatment of SLE patients.

## Author Contributions

ZZ developed the experimental design. ZW wrote the manuscript and prepared the tables and figures. JW, HW, SX, and FZ wrote a part of the manuscript. XX and QT enrolled the patients. YW edited the manuscript. All authors reviewed the manuscript.

### Conflict of Interest Statement

The authors declare that the research was conducted in the absence of any commercial or financial relationships that could be construed as a potential conflict of interest.
